# Anticancer and Cancer Preventive Properties of Marine Polysaccharides: Some Results and Prospects

**DOI:** 10.3390/md11124876

**Published:** 2013-12-02

**Authors:** Sergey N. Fedorov, Svetlana P. Ermakova, Tatyana N. Zvyagintseva, Valentin A. Stonik

**Affiliations:** G.B. Elyakov Pacific Institute of Bioorganic Chemistry, Far-Eastern Branch of the Russian Academy of Science, Prospect 100 let Vladivostoku, 159, Vladivostok 690022, Russia; E-Mails: fedorov@piboc.dvo.ru (S.N.F.); swetlana_e@mail.ru (S.P.E.); zvyag@piboc.dvo.ru (T.N.Z.)

**Keywords:** marine organisms, polysaccharides, anticancer, chemoprevention, anticarcinogenic, mechanisms of action

## Abstract

Many marine-derived polysaccharides and their analogues have been reported as showing anticancer and cancer preventive properties. These compounds demonstrate interesting activities and special modes of action, differing from each other in both structure and toxicity profile. Herein, literature data concerning anticancer and cancer preventive marine polysaccharides are reviewed. The structural diversity, the biological activities, and the molecular mechanisms of their action are discussed.

## 1. Introduction

Polysaccharides are characteristic metabolites of many marine organisms, particularly of algae. Macrophytes such as brown, red, and green algae are known as traditional food ingredients for people populating seaboard geographic areas. In many countries, brown algae belonging to *Laminaria*, *Saccharina*, *Fucus*, *Alaria*, *Sargassum*, *Undaria*, *Pelvetia* genera, green algae such as *Ulva* spp., *Caulerpa lentilifera* as well as red algae such as *Gracilaria* spp., *Porphyra* spp. and others represent an important part of diet, while the purified gelling and thickening ingredients are predominant as food products of algal origin in European countries and the USA. Nowadays, algae have been marketed worldwide as constituents of dietary supplements due to their antimutagenic, anticoagulant, and antitumor properties as well as the high content of so-called dietary fiber.

High content of polysaccharides not only in algae, but also in many other marine organisms, their unusual structures and useful properties make these compounds promising natural products for medicinal and dietary applications, and are utilized in various biotechnologies [1]. Polysaccharides are used in drug compositions, burn dressings, as materials for encapsulation, in various drinks, *etc.* The therapeutic potential of marine polysaccharides enables their utilization for cell therapy and tissue engineering [2].

Many polysaccharides and/or their derivatives such as degraded and semi-synthetic products, obtained by chemical modifications, demonstrate anticancer and cancer preventive properties. They can possess either a direct inhibitory action on cancer cells and tumors or influence different stages of carcinogenesis and tumor development, recover the broken balance between proliferation and programmed cell death (apoptosis) and are useful for cancer prophylactics. Some of these marine natural products have advantages due to their availability, low toxicity, suitability for oral application as well as having a great variety of mechanisms of action [3]. The methods of extraction, fractionation, and purification of polysaccharides from various sources are well known and have been published in many articles [4,5,6,7,8].

Herein, we review some of the literature data concerning anticancer and cancer preventive activity of marine polysaccharides with particular attention to results of the last 10 years.

## 2. Polysaccharides from Brown Algae

### 2.1. Fucoidans

Polysaccharides from brown algae (Phaeophyceae) are well known for their anticancer and cancer preventive properties [9]. These compounds have various important biological functions including a protective role against heavy metal toxicity [10].

**Figure 1:**
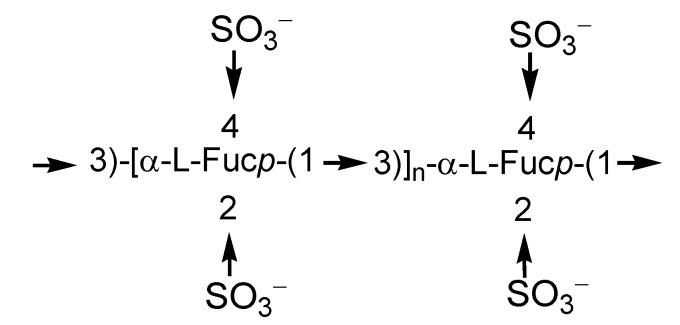
Fucoidan from *Laminaria cichorioides*.

**Figure 2:**
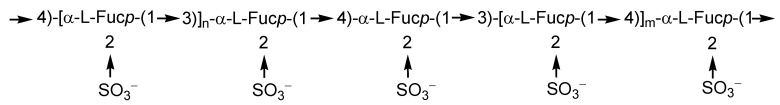
Fucoidan from *F. evanescens*.

Fucoidans can be roughly divided into structural types as follows: α-l-fucans, galactofucans, fucomannouronans and other intermediate structures [11]. Fucoidans isolated from many edible brown algae contain mainly sulfated l-fucose residues attached to each other by α-1,3- or interchangeable α-1,3- and α-1,4-bonds. The regular structures may be masked by random acetylation and sulfation. Some fucoidans have branched structures. As a rule, fucoidans from different algal species differ from each other and vary not only in positions and level of sulfation and molecular mass, but sometimes in the structures of the main carbohydrate chains [12,13]. For example, the fucoidan from the brown alga *Saccharina* (=*Laminaria*) *cichorioides* is 2,4-disulfated 1,3-α-l-fucan (Figure 1), while the fucoidan from *Fucus evanescens* (Figure 2) contains blocks of α-1,3-fucooligosaccharides and α-1,4-fucooligosaccharides sulfated at the position 2 in fucose residues [14,15,16,17].

**Figure 3:**
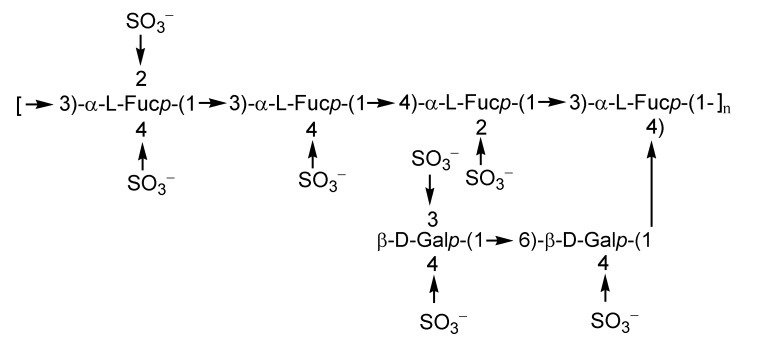
Galactofucan from *L. japonica*.

Galactofucans in contradistinction from α-l-fucans demonstrate considerable structural diversity [18,19]. Sulfated and often acetylated galactofuсans are also widespread in brown algae, including edible ones, such as *Undaria pinnatifida* and *Laminaria japonica*. The main chain of galactofucan can be constructed of blocks or of alternating residues of fucose and galactose. The type of bonds between the monosaccharide residues in galactofuсans, the structure of branchings, the position of sulfates or acetates, as well as the molecular weight can be very multifarious [13,20]. For example the structural fragment of galactofucan from *L. japonica* [21] is provided in Figure 3.

Some brown algae species contain other fucose-embracing heteropolysaccharides such as rhamnofucanes, uronofucanes, *etc.* For example, the main structure of the fucoglucuronomannan from *Kjellmaniella crassifolia* is [-4-d-Glc*p*UAβ1-2(l-Fuc*p*(3-*O*-sulfate)α1-3)d-Man*p*α1-]*_n_* [22]. Uronofucanes are often named as U-fucoidans.

The structural diversity of fucoidans has not yet been sufficiently studied. The structural complexity of fucoidans, the existence of many sub-classes of these glycans in their biological sources as well as a lack of automatic sequencing methods for these polysaccharides have stimulated structure-function studies on the so-called fucanomes in the corresponding marine organisms [23,24]. This research is necessary for the solution of problems of standardization of preparations on the basis of fucoidans, which have attracted attention as practically nontoxic natural products [25,26,27] with antitumor, immunomodulatory, and other useful properties [24,28,29,30].

The anticancer properties of fucoidans have been established many times by *in vitro* and *in vivo* experiments [9,12,13,31,32,33]. It was reported that *Cladosiphon* fucoidan prevented the attachment of *Helicobacter pylori* to the mucin of the gastric tract and, therefore, reduced the risk of associated gastric cancer [34]. While using AGS human gastric adenocarcinoma cells and fucoidan from *Fucus vesiculosus*, it was established that treatment with fucoidan resulted not only in apoptosis of these cells, but also in autophagy with the formation of autophagosomes in fucoidan-treated cells, the conversion of microtubule-associated protein light chain 3 to light chain 3-II and the increase of beclin-1 level [35]. Several reports have also suggested cancer preventive effects of fucoidans on different cellular models. Galactofucan from *U. pinnatifida* inhibited proliferation of prostate cancer PC-3, cervical cancer HeLa, alveolar carcinoma A549, and hepatocellular carcinoma HepG2 cells in a similar pattern to the commercial fucoidan from *F. vesiculosus* [20]. Fucose-containing sulfated polysaccharides from brown algae *Sargassum henslowianum* and *F. vesiculosus* decreased the proliferation of melanoma B16 cells in a dose-response manner. Flow cytometric analysis by Annexin V staining established that both preparations influenced the translocation of membrane phospholipids and activated caspase-3 followed by apoptosis of tumor cells in *in vitro* experiments [36]. Fucoidan from *Ascophyllum nodosum* induced the activation of caspases-9 and -3 and the cleavage of PARP led to apoptotic morphological changes and altered the mitochondrial membrane permeability [37]. Sulfated polysaccharide isolated from the enzymatic digest of *Ecklonia cava* had an effect on caspases-7 and -8 and controlled the cellular membrane molecules Bax and Bcl-xL [38]. The fucoidan from *Sargassum filipendula* showed antiproliferative activity on HeLa cells [39] and induced apoptosis by mitochondrial release of apoptosis inducing factor (AIF) into cytosol, but was not able to activate caspases [40]. The caspase-independent apoptotic pathway was demonstrated for fucoidan from *Cladosiphon novae-caledoniae* [41]. The differences in the mechanisms of apoptosis probably depend upon the structural characteristics of fucoidans and the type of cell lines. Fucoidans were shown to induce apoptosis of some other cancer cells, for example HT-29, HCT116, and HCT-15 human colon cancer cells [42,43] as well as MCF-7 (breast adenocarcinoma) [44], melanoma SK-Mel-28, breast cancer T-47D [45], and human promyeloid leukemic cell lines [46]. MAPK pathways are involved in cellular proliferation, differentiation, and apoptosis induced by fucoidans. The fucoidan from *F. vesiculosus* clearly decreased the phosphorylation of ERKs but not p38 [47]. Another group reported that the pro-apoptotic effect of fucoidan from *F. vesiculosus* was mediated by the activation of ERKs, p38 and by the blocking of PI3K/Akt signaling pathway in HCT-15 cells [42].

Angiogenesis is a multistep process whereby the new blood vessels develop from the pre-existing vasculature. It involves migration, proliferation and differentiation of mature endothelial cells, and is regulated by interactions of endothelial cells with angiogenesis-inducing factors and extracellular matrix components [48]. Fucoidans may suppress tumor growth by inhibiting tumor-induced angiogenesis. Natural and oversulfated fucoidans suppressed the VEGF_165_ induced proliferation and the migration of human umbilical vein endothelial cells (HUVEC) by preventing the binding of VEGF_165_ to its cell surface receptor and inhibiting the VEGF-mediated signaling transduction [49]. In addition, the growth of two types of murine tumor cells inoculated into the footpads of mice was suppressed by administration of natural and oversulfated fucoidans. The relationship between sulfate content in fucoidan from *U. pinnatifida* and the proliferation of human stomach cancer cell line AGS was published [50]. These data showed that antiangiogenic and antitumor activity of fucoidans can be potentiated by increasing the sulfate groups in the molecule [51]. The relationship between the sulfate content of fucoidan and its inhibitory effect on the proliferation of U937 cells was also reported [52]. These results indicated that oversulfated fucoidan induced apoptosis through caspase-3 and -7 activation. The effect of the molecular weight of fucoidan from *U.* *pinnatifida* on the inhibition of cancer cell growth has been investigated. The anticancer activity of fucoidans could be increased by lowering their molecular weight whereby they are depolymerized by mild hydrolysis without a considerable amount of desulfation [53]. The mechanism by which fucoidans inhibited the invasion/angiogenesis of tumor cells has not been clearly elucidated. VEGF is a known angiogenic factor. Fucoidan from *C. novae-caledonia kylin* digested with the abalone glycosidase was responsible for the reduction of MMP-2/9 activities and the decrease in VEGF expression with subsequent inhibition of invasion and suppression of tubules formation in tumor cells [54].

Fucoidans are able to inhibit metastasis of cancer cells. Cell surface receptors belonging to the integrin family have been demonstrated to be involved in the invasion and the metastasis of tumors. The fucoidan from *A. nodosum* inhibited adhesion of MDA-MB-231 (breast adenocarcinoma) cells to fibronectin by binding it and modulating the reorganization of the integrin 5 subunit and down-regulating the expression of vinculin [55].

Cancer preventive properties of fucoidans have been shown in many experiments. For example, decrease of clonogenic growth of tumor cells was demonstrated after treatment with fucoidans [30,45,56]. The inhibition of cell transformation provided evidence on the anti-tumorigenic potential of fucoidans from *A. nodosum* [57], *S. japonica*, *U. pinnatifida*, *Alaria* sp., and *F. evanescens* [29,45,58].

Fucoidans may enhance the anticancer action of some low molecular weight compounds. For example, the fucoidan from the Far-eastern brown seaweed *F. evanescens* at a concentration of 500 μg/mL was not cytotoxic in human malignant lymphoid MT-4 or Namalwa cells. Pretreatment of MT-4, but not Namalwa cells with fucoidan followed by the exposure to DNA topoisomerase II inhibitor etoposide led to about a two-fold increase in the relative apoptotic index as compared with etoposide itself [56]. The fucoidan from *S. cichorioides* enhanced the antiproliferative activity of resveratrol at nontoxic doses and facilitated the resveratrol-induced apoptosis in the HCT116 cell line. Furthermore, the cells were sensitized by the fucoidan to the action of resveratrol and the inhibition of HCT116 clonogenic capacity was indicated [59].

Some fucoidans showed cytoprotective properties. It is important that fucoidan may be useful for the recovery of 5-fluorouracil (5-FU)-treated antigen-presenting dendritic cells, because this clinical anticancer agent induces immunosuppression in cancer patients as a side effect [60].

In the majority of cases, molecular mechanisms of anticancer and cancer preventive actions of fucoidans were established by *in vitro* studies. Many fucoidans induced apoptosis of tumor cells through activation of the caspases and by enhancing mitochondrial membrane permeability. Sometimes this mechanism involved the reactive oxygen species (ROS)-dependent JNK activation as was shown for partly digested fucoidan from commercially available seaweed *C. novae-caledoniae* using MCF-7 and MDA-MB-10A tumor cells [41].

Fucoidans modulate the immune system and may induce functional maturation of human monocyte-derived dendritic cells (DC) [61]. Ligand scavenger receptor class A (SR-A) indirectly participates in maturation of human blood dendritic cells via production of tumor necrosis factor followed by stimulation of T-cells. Thereby, fucoidan acts as a scavenger receptor agonist and maturation is eliminated by pretreatment with TNF-neutralizing antibodies [62]. At a later date, it was confirmed that SR-A plays a crucial role in affecting the DC-mediated presentation of cancer antigens to T cells in human cancer cells, and it was also established that fucoidan promoted the DCs maturation. The fucoidan-treated DCs stimulated the CD8^+^ T limphocytes to release more interferon-γ than non-fucoidan-treated cells. It was found that fucoidan enhanced the cross-presentation of NY-ESO-1 cancer testis antigen to T cells and it led to the increase of T-cell cytotoxicity against NY-ESO-1 human cancer cells [63]. Cytotoxic activities of natural killer cells were also activated *in vivo* after administration of fucoidans from *Sargassum* sp. and *F. vesiculosus* to mice [36].

Fucoidan from *F. vesiculosus* inhibited the migration and the invasion of human lung cancer cells decreasing the cytosolic and nuclear levels of kappa-B nuclear factor [64]. Treatment of mouse breast cancer cells with fucoidan showed that the enhanced antitumor activity was associated with decreased angiogenesis via the down-regulation of vascular endothelial growth factor and increased induction of apoptosis [65].

It has been suggested that the anticarcinogenic action of fucoidan from *S. cichorioides* is connected with its ability to interact directly with epidermal growth factor (EGF) and prevents its binding to EGF receptor (EGFR). Actually, in experiments with neoplastic transformation of JB6 mouse epidermal cells induced by EGF or 12-*O*-tetradecanoylphorbol-13 acetate, a Russian-Korean group of scientists reported that inhibition of EGFR phosphorylation was followed by inhibition of the activities of some extracellular signal regulated kinases that resulted in the inhibition of AP-1 nuclear factor transactivation [66,67].

Ultraviolet irradiation is known to induce skin aging and cause skin cancer. UVB stimulates the activation of cellular signaling transduction followed by the production of metalloproteinases (MMPs). Fucoidans suppressed the UVB induced MMP-1 expression and inhibited ERKs activity in human skin fibroblasts in a dose-dependent manner. They inhibited significantly MMP-1 promoter activity and increased type I procollagen mRNA and protein expression. It was concluded that *Costaria costata* fucoidan may be considered as a potential agent for the prevention and treatment of skin photoaging [68,69,70]. The fucoidan from *F. vesiculosus* post-translationally regulated MMP-9 secretion from human monocyte cell line U937 [71].

Thus, the molecular mechanisms of anticancer and cancer preventive actions of fucoidans are rather complicated and may include inhibitory effects against cancer cell proliferation and induction of tumor cells apoptosis. In addition, these polysaccharides stimulate immunity and inhibit angiogenesis. The cancer preventive action of fucoidans includes such useful properties as anti-inflammatory, anti-adhesive [72], antioxidant and antiviral effects [73,74,75,76] as well as their capability to bind heavy metals. Moreover, these compounds may delay and decrease the action of such factors of carcinogenesis as some tumor promoters (EGF, phorbol esters), defend against UV radiation and inhibit the tumor invasion by modulation of metalloproteinases. Possibly, these effects depend on the differences in the structures of fucoidans isolated from various biological sources and on their physico-chemical characteristics such as molecular weight.

Daily consumption of fucoidan-containing algae was proposed as a factor in the lowering of postmenopausal breast cancer incidence and mortality. Urinary human urokinase-type plasminogen activator receptor concentration is higher among postmenopausal women breast cancer patients. It was shown that this concentration was decreased by about 50% after seaweed supplementation [77]. In addition, fucoidans reduced the toxicity of chemotherapy for patients with unresectable advanced or recurrent colorectal cancer. Fucoidan may enable the continuous administration of such drugs as oxaliplatin plus 5FU/leucovorin and, as a result, may prolong the survival of patients [78]. In some countries food supplements and drinks containing fucoidans are used to treat patients having different cancers. In many countries fucoidan-containing extracts are used as a remedy in traditional medicine.

In our opinion, the perspectives of studies on fucoidans are connected with further search for new structural variants of these types of polysaccharides and the relationships established between the structures and the biological activities. The great diversity of fucoidans, presenting in brown algae and covering a much broader range than only those having a fucan backbone, provides potential for the future discovery of numerous new polysaccharides of this class and their derivatives. Fucoidan bioactivities depend on the extraction and the purification methods used, because fucoidans obtained from the same biological source using different methods differ from each other in the content of sulfate groups and in the impurities [79]. Furthermore it is known that the content and structure of fucoidans depends on the seaweed species, the parts of the plant, the harvest season and mainly on the stage of development of the algae [58,80,81].

The recent rapid progress in studies on fucoidans has been achieved by application of modern methods of structural investigation such as 2D NMR, MALDI-TOF and tandem ESI mass-spectrometry [82,83] as well as new techniques of molecular biology and pharmacology such as fluorescent staining, flow cytometry, *mi*-RNA, Western blot, *etc.*

### 2.2. Laminarans

**Figure 4:**
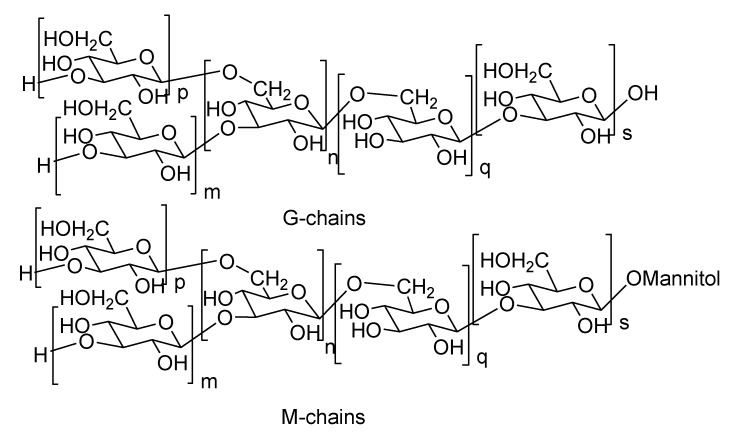
The structures of G- and M-chains of laminarans.

Important results have been obtained in the studies on other algal polysaccharides from brown algae laminarans, as potential cancer preventive agents. Laminarans are low molecular weight polysaccharides (MW about 3–6 kDa) consisting mainly of 1,3-linked β-d-glucopyranose residues with a small number of 1,6-bonded β-d-glucopyranose units in the main and the branching chains. Their carbohydrate chains are terminated with d-mannitol residues (so-called M-chains) or contain glucopyranose residues only (so-called G-chains) (Figure 4). Sometimes terminal residues of M-chains may be additionally glycosylated or M-chains may be completely absent [84]. Branching at positions 2 and 6 was found in the laminaran from *Saccharina longicrucis* [85].

High molecular weight laminaran (19–27 kDa) was recently isolated from the brown seaweed *Eisenia bicyclis.* It was shown that this 1,3;1,6-β-d-glucan contained 1,6-linked glucose residues in both branches and the main chain, basically in the non-reduced ends of the molecules. This laminaran and its products of enzymatic degradation inhibited the colony formation of SK-Mel-28 and colon cancer DLD cells. The increase of the content of 1,6-linked glucose residues and the decrease of the molecular weight improved the anticancer effect in this series of substances [85]. It is known that algal glucans suppress angiogenesis in tumor growth. Recent findings show that they enhanced the tumor response to photodynamic therapy in C57BL/6 mice, administered subcutaneously with Lewis lung carcinoma cells. Ten days after implantation, the mice were treated with sodium porfimer, 24 h prior to laser irradiation with or without oral administration of β-d-glucans. When algal β-d-glucan was used, significantly reduced tumor growth was indicated [86].

Laminarans noticeably inhibited the formation of putrefactive and harmful compounds, such as indoles, p-cresol, ammonia, phenol, and sulfide, produced by the fecal microflora. These putrefactive compounds in rats fed low molecular alginate also tended to be lower. In both experiments (with laminaran and with alginic acid) the intestinal bacterial flora of rats was changed. Polysaccharides were fermented into propionic and butyric acids by intestinal microbiota, similar to the effects of prebiotics. These results suggest that the fermentation of laminaran by intestinal bacteria could suppress the risk of colorectal cancer [87,88]. It is of special interest that not only laminarans, but also other β-d-glucans, isolated from yeast, fungi and cereals demonstrated anti-cytotoxic, anti-mutagenic, and anti-tumorigenic properties, making this class of polysaccharides a promising promoter of health [89].

Tumor metastasis is connected with expression of heparanase, an endo-β-d-glucuronidase that degrades the main polysaccharide constituent of the extracellular matrix and the basement membrane. In fact, expression of the heparanase gene is associated with the invasive potential of tumors. Laminaran sulfate inhibited heparanase enzymatic activity and reduced the incidence of metastasis in experimental animals [90].

### 2.3. Alginic Acids

**Figure 5:**
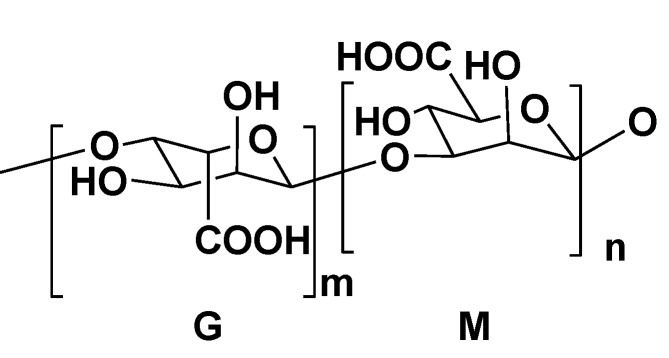
Structure of alginic acid.

Alginic acids are widely distributed in the cell walls of brown seaweeds. These anionic polysaccharides were proved to be linear polymers containing blocks of 1,4-linked β-d-polymannouronate and α-l-polyguluronate (so-called M- and G-blocks) (Figure 5). Molecular masses of alginic acids ranged between 10 kDa and 600 kDa. These polysaccharides are used in the pharmaceutical industry and in biotechnology, particularly for cell immobilization and encapsulation.

Alginic acid-coated chitosan nanoparticles have been constructed as an oral delivery carrier for the legumain-based DNA vaccine. It was shown that this vaccine could effectively improve autoimmune response and protect against breast cancer in mice [91].

Biopreparations containing alginic acids probably have some cancer preventive properties because of the ability of polysaccharides to bind toxins and heavy metals in the intestines and transform these dangerous compounds into less harmful forms.

## 3. Polysaccharides from Red Algae

Red algae (Rhodophyta) contain several classes of well known polysaccharides, having wide application in microbiology, biotechnology and other fields, mainly due to the ability of their aqueous solutions to form strong gels. Sulfated galactans such as agar, agarose and carrageenans usually contain repeating disaccharides of β-(1→3)-linked and α-(1→4)-linked galactopyranosyl (Galp) residues. Several red algae species contain other polysaccharides, for example mannans and xylans [92].

**Figure 6:**
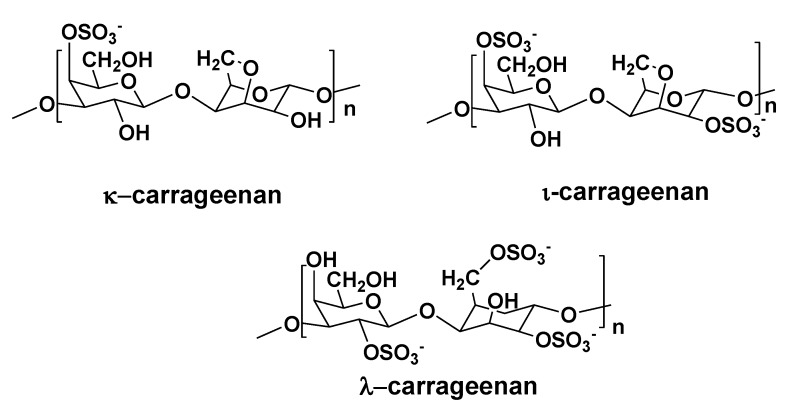
Structures of repeating units of some carrageenans.

All carrageenans consist of either galactose or galactose and 3,6-anhydrogalactose monosaccharide units and differ from each other in monosaccharide composition, level of sulfation, positions of sulfate groups and molecular weights. Three groups of carrageenans, so-called kappa-, iota- and lambda-carrageenans, are of commercial significance (Figure 6). Hybrid forms of carrageenans are also known.

Some representatives of this polysaccharide class demonstrate properties connected with cancer prevention, mainly due to antiviral, antioxidant properties, and stimulation of antitumor immunity. As is known, certain sexually transmitted human papillomavirus types are associated with the development of cervical cancer. Recently, it was established that carrageenan in nanomolar concentrations inhibits papillomavirus. However, clinical trials are needed to determine, whether carrageenans are effective as antiviral drugs against genital human papilloma viral infection or not [93].

κ-Carrageenans degraded by an oxidative method involving hydrogen peroxide (H_2_O_2_) treatment were evaluated as scavengers of superoxide anions and hydroxyl radicals by application of flow injection chemiluminescence technology. The values of IC_50_ of degraded κ-carrageenans labeled A, B, C, and D against the superoxide anion showed a positive correlation with molecular weight. As for hydroxyl radical scavenging, the EC_50_ values of degraded κ-carrageenans A, B, C, and D showed the same correlation. Therefore, these results indicated that κ-carrageenans with lower molecular weights have better antioxidant properties and may be promising for cancer prevention [94]. Carrageenan oligosaccharides from the red alga *Kappaphycus striatum* were perorally administrated during 14 days into mice inoculated with S180 tumor cell suspension. This resulted in growth inhibition of transplantable sarcoma cells, increased macrophage phagocytosis, enhanced antibody production, increased lymphocyte proliferation, stronger NK cell activity, and elevated levels of IL-2 and TNF-α. These results suggested that the studied oligosaccharides exert their antitumor effects by promoting the immune system [95]. *In vivo* antitumor activities for κ-carrageenan oligosachharides and low molecular λ-carrageenan from *Chondrus ocellatus* have been established. The latter also potentiated the antitumor effect of 5-FU [96,97]. Similar data were obtained in studies of sulfated polysaccharide from the red alga *Champia feldmannii* [98]. Thus, low molecular carrageenans and carrageenan oligosaccharides seem to be more promising cancer preventive agents than high molecular natural products belonging to this class of polysaccharides.

However, harmful gastrointestinal effects of both native and degraded carrageenans followed by the induction of neoplasms in animal experiments were reported [99]. Later, it was confirmed that degraded carrageenan induces colitis in rats *in vivo* and induces inflammation. However, in experiments *in vitro*, the preparation inhibited proliferation of THP-1 cells and arrested the cells in the G1 phase [100]. In another review concerning the toxicological effects of carrageenan on the gastrointestinal tract, it was demonstrated that systematically perorally administrated carrageenan was not carcinogenic. It was noted that previous toxicological studies involved administration of doses that exceeded those to which humans are exposed by several magnitudes [101]. Similar conclusion about the safety of peroral application of κ-carrageenan was made as a result of a 90-day dietary study in rats [102].

Thus, further investigations are needed to determine the applicability of partly degraded carrageenans as cancer preventive agents.

## 4. Polysaccharides from Green Algae

**Figure 7:**
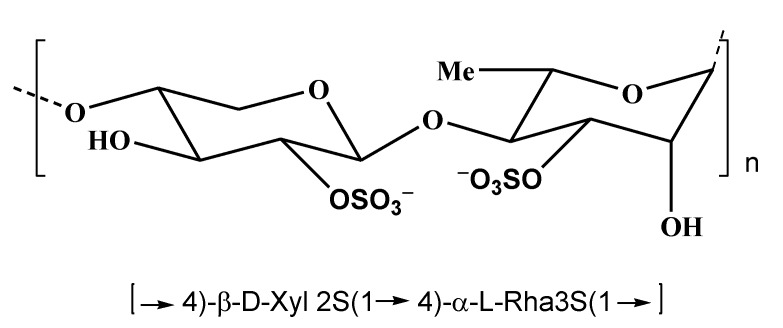
Structure of the main repeating disaccharide in *Ulva* *rigida*.

Among marine macrophytes, marine green algae have been less studied in comparison to brown and red algae as sources of polysaccharides with anticancer and cancer preventive properties. However, their antitumor properties have been sometimes reported, mainly for the polysaccharides belonging to the so-called ulvans. Ulvans, water soluble sulfated polysaccharides from the cell walls of green algae are characteristic of the plants, belonging to the genera *Ulva*, *Enteromorpha*, *Monostroma*, *Caulerpa*, *Codium*, and some others. They are composed of repeating disaccharide moieties, containing sulfated rhamnose and uronic acid (glucuronic or iduronic). The structure of the disaccharide moieties of ulvans resembles that of glycosaminoglycans, which occur in the extracellular matrix of connective tissues of animals. Some ulvans include also xylose residues (Figure 7) [103].

The highly pyruvated 1,3-β-d-galactan sulfate from the Pacific *Codium yezoense* and the similar polysaccharide from *Codium isthmocladium* represent another type of polysaccharides found in green algae [104,105]. Sulfated β-d-mannans like that isolated from *Codium vermilara* [106] have also been found.

Promising antioxidant and antiproliferative activities were recently found in the sulfated polysaccharides isolated from several tropical species of green algae. HeLa cell proliferation was inhibited between 36.3% and 58.4% after 72 h incubation with the polysaccharide isolated from *Caulerpa prolifera* [107]. Two polysaccharide fractions obtained from the green alga *Caulerpa racemosa* showed antitumor activities, and their inhibition rates of H22 tumor transplanted in mice were 59.5%–83.8% (48 h) and 53.9% (14 days) at a dose of 100 mg/kg/day, respectively [108].

*In vivo* and *in vitro* stimulation of immunity was indicated as the action of water-soluble sulfated polysaccharide fractions from *Enteromorpha prolifera*. These polysaccharides significantly increased ConA-induced splenocyte proliferation and induced the production of various cytokines via up-regulated m-RNA expression [109]. The ulvan from *Ulva rigida* induced more than a two times increase in the expression of some cytokines, stimulated the secretion and activity of murine macophages as well as inducing an increase in COX-2 and NOS-2 expression [110]. Ulvans from *Ulva pertusa* had little cytotoxicity against tumor cells, but significantly stimulated immunity, inducing considerable amounts of nitric oxide and cytokine production [111]. There are several reports concerning the antioxidant activities of ulvans in experimental D-galactosamine-induced hepatitis in rats [112,113].

The strong immuno-modulatory potencies as well as the antioxidant properties of polysaccharides from green algae suggest their potential cancer preventive activity and their future utilization as experimental immuno-stimulants.

## 5. Polysaccharides from Microalgae

There is little information concerning cancer preventive and anticarcinogenic properties of polysaccharides from marine microalgae, although these organisms have been used for a long time as food for humans, particularly *Arthrospira* (the former name *Spirulina)* and *Porphyridium*. Similar marine organisms belong to the classes Bacillariophyceae (diatoms), Cyanophyceae (blue-green algae), Porphyridiophyceae and partly to Chlorophyceae and Rhodophyceae. However, after the nuclear accident of Fukushima and the resulting radioactive pollution, the ability of marine algae to bio-accumulate radionuclides, has become a major concern. For example, the newly discovered green microalga, *Parachlorella* sp. *binos* (Binos) exhibited highly efficient incorporation of radioactive isotopes of iodine, strontium and cesium. The authors also showed the ability of microalgae to accumulate radioactive nuclides from water and soil samples collected from the heavily contaminated area in Fukushima [114]. Determination of the potential radioactive contamination of seaweeds is therefore crucial before further search for bioactive compounds. Polysaccharides isolated from various microalgae ranging from diatoms to green-blue algae demonstrated different activities, although direct anticancer properties were rarely reported [91]. Apoptogenic properties of red microalgal polysaccharides in two human tumor cell lines MCF-7 and HeLa were established [115]. Some microalgal polysaccharides were found to show antiviral activities against retroviruses. These viruses, containing reverse transcriptase are implicated in various types of leukemias and other tumors. Polysaccharides from the fresh water red microalga *Porphyridium* sp. were more active than those from *Porphyridium aerogineum* and *Rhodella reticulata* against murine leukemia virus (MULV) and murine sarcoma virus (MuSV-124) in cell culture [116]. Marine red microalgae polysaccharides and polysaccharides from other microalgae were also studied in this respect. For example, sulfated polysaccharides from the marine microalga *Cochlodinium polykrikoides* showed a significant *in vitro* antiviral activity against human immunodeficiency virus and absence of a cytotoxic effect directed against the host cells [117]. Antiviral properties were found in several other polysaccharides, isolated from different microalgae [118,119].

In addition, blue green algal polysaccharides were immuno-active and showed antioxidant and free radical scavenging properties [115]. High molecular weight polysaccharides from the fresh water *Spirulina* *platensis* and related species [120] were between one hundred and one thousand times more immuno-active than polysaccharide preparations from other biological sources that are used clinically for cancer immunotherapy. Actually, related compounds with similar properties should be found in the corresponding marine species. Antioxidant activity was also reported for polysaccharides from *Porphiridium cruentum* [121].

All these activities are usually associated with anticancer and cancer preventive properties. For example, it is known that oxidative stress can lead to cancer and some antioxidant marine products proved to be chemopreventive antitumor agents [122]. Cancer preventive action of the oligosaccharide derived from the microalga *P. cruentum* was reported [123]. Another example concerns the extract from the deep-sea water *Spirulina maxima*, which effectively suppressed the expression of Bcl2 in A549 cells and inhibited viability of other human cancer cells [124]. *Spirulina platensis* preparations showed the chemopreventive effect against carcinogenesis induced by dibutyl nitrosamine with the decrease of the incidence of liver tumors from 80% to 20%. However, it is unknown, whether polysaccharide contribution is significant in this case or not [125].

## 6. Polysaccharides from Marine Bacteria and Fungi

A great diversity of polysaccharides from marine bacteria and fungi also attract attention because of their structures, anticancer and cancer preventive properties. Polysaccharide B1 from the marine *Pseudomonas* sp. has repeating units as -2)-β-d-Gal*p*(4-sulfate)(1,4)[β-d-Glc*p*(1,6)]-β-d-Gal*p*(3-sulfate)(1- and demonstrated cytotoxicity against tumor cells, being more active to the central nervous system and lung cancer cell lines. It induced apoptosis in U937 cells [126].

The marine filamentous fungus *Keissleriella* sp. YS 4108 polysaccharide with a mean molecular weight of 130,000 Da showed radical eliminating and antioxidant actions in various *in vitro* systems. In addition to scavenging activities, the polysaccharide effectively blocked the non site-specific DNA strand-break induced by the Fenton reaction at concentrations of 0.1 and 1 mg/mL. These results suggested that this preparation could be of preventive and therapeutic significance to some life-threatening health problems such as cancer [127].

## 7. Polysaccharides from Marine Animals

Polysaccharides can be found in various marine animals such as sea cucumbers, sea urchins, sponges, starfish, ascidians, *etc.* They contain a great variety of polysaccharide compounds, including glycosaminoglycans, fucans, and galactans [23,128,129,130]. These compounds demonstrate diverse biological properties, including anticoagulant and antitrombotic [131,132,133], antioxidative [134], neuroprotective [135,136], and antiviral activity as well [8,137]. However, anticancer and cancer preventive activities of the polysaccharides from marine animals have been studied insufficiently. Polysaccharide SEP isolated from the eggs of the sea urchin *Strongylocentrotus nudus* effectively inhibited the growth of S180 tumor and the hepatocellular carcinoma * in vivo* via the activation of lymphocytes and macrophages, amplification of B and T cell proliferation, and increased secretion of such cytokines as IL-2, TNF-α and IFN-γ [138,139,140,141]. The sulfated polysaccharide conjugate from viscera of abalone *Heliotis discus hannai*, administered at doses of 1–40 mg/kg to mice inhibited tumor growth and increased lymphocyte proliferation, as well as natural killer cell activity and antibody production. A significant increase of immune function was observed in cyclophosphamide-induced immunosuppressive mice on administration of 40 mg/kg dose [142].

Cancer chemoprevention implies the use of natural or synthetic compounds for prevention, suppression or reversal of the process of carcinogenesis [143]. Cancer preventive compounds may stimulate anticancer immunity, inhibit inflammation, angiogenesis and tumor invasion, or protect from UV-radiation damage [144,145,146,147]. Preincubation with mytilan, a polysaccharide isolated from the mussel *Crenomytilus grayanus*, was followed by a normalization of the activity indicators of human peripheral blood lymphocytes and by a reduction of the number of morphological defects of the marine invertebrates larvae after UV-irradiation [148]. Sulfated polysaccharide obtained from the sea cucumber *Cucumaria frondosa* affected the maturation of monocyte-derived dendritic cells and their activation of allogeneic CD4(+) T cells *in vitro* by down regulation of the secretion of IL-10 and IL-12p40 at 100 μg/mL [149]. Some polysaccharides from the marine animals inhibited the binding of pro-inflammatory molecules, P- and L-selectins, to immobilized carbohydrate determinant sialyl Lewis^x^ which is a component of cell surface glycoproteins presented in leukocytes and overexpressed in several tumor cells. As a consequence of their antiselectin activity, these polysaccharides attenuated metastasis and inflammation [150,151,152]. Oral administration (100 mg/kg body weight) for five days of sea cucumber fucoidan (SC-FUC) extracted from *Acaudina molpadioides* can significantly prevent the formation of gastric ulcer in rats. Moreover, SC-FUC pretreatment could alleviate ethanol-induced histological damage, reverse changes in tissue oxidation and antioxidase activities, and regulate the signaling pathways of mitogen-activated protein kinases and matrix metalloproteinases [153]. Chondroitin sulfate isolated from ascidian *Styela clava* inhibited phorbol ester- and TNF-α-induced expression of inflammatory factors VCAM-1, COX-2 and iNOS by blocking Akt/NF-κB activation in mouse skin [154,155]. Anti-inflammatory activity of heparin analogues from ascidians and marine shrimps was also reported [156,157]. The heparin isolated from white leg shrimp demonstrated anti-angiogenic activity [158].

## 8. Conclusion

To date, numerous polysaccharides have been isolated from different marine organisms ranging from marine bacteria to marine animals and several dozen of them have attracted attention as promising anticancer and cancer preventive substances. Some of these compounds are already used in clinical practice. Polysaccharide anticancer and cancer preventive substances demonstrate a wide variety of useful properties and mechanisms of action, including inhibition of tumor cell proliferation, induction of apoptosis, inhibition of angiogenesis, *etc.* These biopolymers and their derivatives frequently show radical scavenging, antiviral, and immuno-stimulatory properties. Polysaccharides obtained from marine invertebrates possess unique physico-chemical and biological properties, which justify intensive research efforts in the future. The increasing exploration of marine biological sources will help to identify the most promising of these compounds.
